# Is bone loss a physiological cost of reproduction in the Great fruit-eating bat *Artibeus lituratus*?

**DOI:** 10.1371/journal.pone.0213781

**Published:** 2019-03-28

**Authors:** Diego A. Torres, Mariella B. Freitas, Sérgio L. P. da Matta, Rômulo D. Novaes, Reggiani Vilela Gonçalves

**Affiliations:** 1 Animal Biology Department, Federal University of Viçosa, Viçosa, Brazil; 2 General Biology Department, Federal University of Viçosa, Viçosa, Brazil; 3 Structural Biology Department, Federal University of Alfenas, Alfenas, Brazil; Montana State University, UNITED STATES

## Abstract

During mammalian pregnancy and lactation, the maternal demand for calcium is increased to satisfy fetus and newborn skeletal growth. In addition to the dietary intake, females use the calcium contained in their bones to supply this increased demand, leading to a decrease in maternal bone mineral content. In reproductive insectivorous female bats, bone loss has been described as a physiological cost of reproduction, due to the reported increased risk of bone fracture. This physiological cost may be the mechanism underlying the conflict between increasing litter size and maintaining wing skeletal integrity, which would help to explain the small litter size of most bat species. If bone loss is a linking cost between reproduction and survival in bats, and most bat species have small litter sizes, one would expect to find a loss of bone and an increasing probability of bone fracture during pregnancy and lactation in other non-insectivorous bats. In this study, we tested for the existence of this cost in the Great-fruit eating bat, *Artibeus lituratus*. We analyzed trabecular structure, bone strength and bone mineral content for the humerus bone, hypothesizing that bone loss during reproduction in females would increase the risk of fracture. Our results showed a decrease of 22–31% in bone trabecular area in lactating females, rapidly compensated following weaning. Bone strength did not differ among reproductive and non-reproductive groups and seems to be more influenced by bone organic components rather than mineral contents. Since we observed bone loss during reproduction yet the humerus strength seems to be unaffected, we suggest that bone loss may not represent a physiological cost during reproduction for this frugivorous bat.

## Introduction

Size at birth, age at maturity or growth patterns are life-history traits that contribute to the strategy of an organism to successfully achieve survival and reproduction [1]. However, it has been proven that these traits cannot be selected simultaneously to increase fitness’ components [2,3], therefore organisms must trade-off between different traits (e.g. number of offspring vs. size of offspring [4]).

Bats are the second largest group of mammals in terms of species number and despite their physiological and ecological diversity, they exhibit little variation in their litter size [5]. Most bats deliver one pup per litter, a remarkable pattern considering that most bats have small body sizes. Other mammals of similar body size, such as rodents or shrews, deliver larger litters and produce more offsprings per year than do bats [5,6]. Additionally, young bats are nutritionally dependent on their mothers for a long period of time, due to their need to reach 70–80% of adult size to withstand the stress of flight [7], while other terrestrial mammals of similar size are nutritionally independent when they reach about 40% of their adult size [6]. This implies that female bats invest more energy and nutrients during lactation in each young than terrestrial mammals of similar size, which may limit large litter sizes in bats.

The nutritional investment and the use of bone calcium in reproductive bats is reflected in the reduction of the total body calcium, pup body calcium, and litter calcium content during lactation in *Eptesicus fuscus* [8]. Similar patterns have been observed in other insectivorous bats such as *Myotis lucifugus* [9,10] and *Miniopterus schreibersii* [11], which lose bone calcium from the humerus, mandible and femur during lactation. The loss of bone in insectivorous bats during reproduction probably decrease their fitness due to mineral loss and its consequent decrease in bone strength, which increases the risk of fracture [12,13]. This would impair the effectiveness of foraging and the ability to avoid predators, which could impact survivorship and the chances for future reproduction [10,14]. If pregnancy and lactation in bats decrease the probability of survival due to bone loss during reproduction, the small litter size exhibited by bats may be the result of a trade-off between increasing litter size and skeleton integrity [6,10].

If bone loss is a physiological cost linking the trade-off between reproduction (litter size) and survival (skeleton integrity) in bats, and most bat species have small litter sizes, one would expect to find bone loss and an increased risk of fracture during pregnancy and lactation in other, non-insectivorous bats. Here we investigated whether patterns of bone loss in a frugivorous bat species is similar to that of insectivorous bats, and whether pregnancy and lactation result in decreased bone mineral content and biomechanical performance. We tested this hypothesis for the Great-fruit eating bat (*Artibeus lituratus*), a Neotropical bat species that plays a role on forest regeneration through seed dispersal. For this analyses, we selected the humerus, the most calcified bone in the bat wing [15], hypothesizing that reproductive females would show a decrease in bone area, calcium content and strength parameters compared to non-reproductive females.

## Materials and methods

### Bat sampling and tissues processing

Adult females (*A*. *lituratus*, n = 31) were captured using mist-nets at night in two fragments of Atlantic Forest (fragment 1: 20°45’22.89”S 42°51’48.1”W; fragment 2: 20°48’09.5”S 42°51’32.45”W) from October 2015 to September 2016, in Viçosa, MG, Brazil. Captured females were taken to the Laboratory of Experimental Pathology of the Federal University of Viçosa for tissue collection. All bats were euthanized in the morning following capture by decapitation, after an intraperitoneal injection with xylazine (10 mg/kg) and ketamine (60 mg/kg). [16]. National environmental authorities approved animal capture and transportation (license number 50517–1 SISBIO) and the Animal Use and Care Committee of the Federal University of Viçosa approved all the procedures and protocols used (CEUA/UFV–number 89/2015).

To ensure adult status, only animals with complete ossification of the cartilaginous epiphyseal growth plates of metacarpal phalangeal joints were selected [17]. Females were assigned to one of the following groups: Pregnancy (P, n = 8), when a fetus was identified by abdominal palpation; Lactation (L, n = 8), when the nipples were producing milk and the area around the nipple lacked fur; Post-lactation (PL, n = 6), when nipples were similar to those of lactation but were not producing milk and the area around the nipple was recovering its fur; and Non-pregnant (NP, n = 9), when the female was not assigned to the first three categories. Non-pregnant status was confirmed by dissection of the uterus. We decided to use Non-pregnant females as controls since they go through the same reproduction-related cycles of losing and gaining bone tissue, therefore the comparison of reproductive vs non-reproductive females was considered more appropriated to test our hypothesis.

In all groups, both humeri were extracted, cleaned and weighted. The left humerus was wrapped in gauze, soaked in saline solution and stored at -20°C for bone strength analysis [18]. The proximal portion of the right humerus was fixed in neutral buffered formalin for 48 h and then transferred to ethanol (70%) for histological processing and subsequent morphometric analyses [19].

### Bone histomorphometric analyses

Proximal portions of the humerus were immersed in a formic acid (12.5%) and sodium citrate (20%) decalcifying solution for 30 days, both mixed in equal parts. Decalcified bones were cut longitudinally to expose the trabecular bone of the epiphysis. Bone fragments were then dehydrated in ethanol, cleared in xylol, embedded in paraffin and cut in sections of 5-μm-thick histological sections. Sections were stained with hematoxylin and eosin [20]. We randomly selected and digitized 10 regions of interest (ROI) in each female using the 10x objective lens in light microscope (Olympus BX-60, Tokyo, Japan) integrated with a digital camera (Olympus QColor-3, Tokyo, Japan). Each ROI represented an area of the 36x10^4^ μm^2^ of trabecular bone. We deleted the marrow in each ROI in order to estimate bone area (B.Ar), considering only the trabeculae, and transformed the ROI in a black and white image (trabeculae was black and the background was white). Then, we calculated how much of the ROI were covered by trabeculae [21]. Black and white images were also used to estimate trabecular width (Tb.Wi) and trabecular separation (Tb.Sp) using the spheres fitting method. This method uses the average of the diameters of all spheres that can be fit in the trabeculae (Tb.Wi) or between the trabeculae (Tb.Sp) [22]. The greater the diameter of the spheres fitted on the trabeculae, the greater the width of the trabeculae; and the greater the diameter of the spheres fitted between trabeculae, the smaller the area occupied by trabeculae (S1 Fig). Abbreviations of bone histomorphometric parameters follow the Standardized Nomenclature, Symbols, and Units of the American Society for Bone and Mineral Research [23]. Analyses were run using the software ImageJ [24] with the plugin BoneJ [25].

### Bone strength test

Left humeri were taken from -20°C and left at room temperature three hours prior to testing. A three point bending test was performed using a universal testing machine system (UTM, Instron 3367). We applied a force in the middle third (diaphysis) of the humerus with a load cell of 250 N (Newtons) at a speed of 10 mm/s until complete fracture. The maximum load and maximum displacement were obtained directly from the UTM. These data were used to calculate the extrinsic stiffness, using the slope in the linear region of the elastic region of the load-displacement curve [26].

### Bone mineral content analysis

After bone strength test, left humeri were dried at 70°C until they reached constant weight. Bones were homogeneously macerated and 2 g of each bone were digested in 5 mL solution containing 2,5 mL of nitric acid and 2,5mL of perchloric acid at 200°C. Then, we completed to a volume of 10 mL with distilled water and determined the concentrations of calcium and phosphorus by atomic absorption spectrometry (Varian SpectrAA Model 220FS) [27].

### Statistical analysis

We calculated the mean ± standard deviation for all variables for each group. Shapiro-Wilk and Levene’s tests were used to test normality and heteroscedasticity, respectively (S1 File). Since all variables were normally distributed and not heteroscedastic, we performed a one-way Analysis of Variance (ANOVA) to test differences between reproductive groups, followed by the Tukey test to compare all pairs. Simple linear regressions were used to test the relationship between variables. ANOVAs were run in the statistical software R, through R commander package [28], and regressions were run in GraphPad Prism (Version 5.04, GraphPad Software, La Jolla California USA) (26). The significant level was set at 5% (p<0.05). Dataset is full available as S2 File.

## Results

Body weight of pregnant females was higher than in non-pregnant and lactating females, but not significantly different from post-lactating females (Table 1). Considering the macroscopic and microstructural parameters analyzed, post-lactating bats showed increased values for bone dry weight as compared to all other groups, including the non-pregnant group (Table 1). Bone trabecular area was higher in pregnancy and post-lactation groups compared to lactating bats, indicating reduced bone mass in lactating bats (Table 1). All other parameters showed similar values among reproductive groups (Table 1).

**Table 1:** Macroscopic and microstructural parameters in female bats (*Artibeus lituratus*).

	Reproductive groups					
	Non-pregnant	Pregnancy	Lactation	Post-lactation	F	*P*
Body weight (g)	69.40 ± 8.30^**A**^	79.77 ± 7.05^**B**^	66.97 ± 6.67^**A**^	71.74 ± 1.61^**AB**^	5.485	**0.004**
		
B. length (mm)	43.01 ± 1.35	42.49 ± 1.35	42.63 ± 1.96	42.94 ± 0.61	0.234	0.872
B. wet weight (g)	0.54 ± 0.05	0.56 ± 0.05	0.53 ± 0.06	0.58 ± 0.05	0.978	0.418
B. dry weight (g)	0.31 ± 0.02^**A**^	0.36 ± 0.03^**AB**^	0.32 ± 0.04^**AB**^	0.38 ± 0.05^**B**^	3.766	**0.022**
B.Ar (%)	29.58 ± 4.6ab^**AB**^	30.09 ± 6.58^**B**^	23.29 ± 4.20^**A**^	33.95 ± 3.20^**B**^	5.824	**0.003**
Tb.Wi (μm)	76.42 ± 19.54	73.42 ± 15.18	60.59 ± 26.45	79.26 ± 13.87	1.241	0.314
Tb.Sp (μm)	207.06 ± 30.15	196.91 ± 60.87	226.86 ± 26.45	187.06 ± 38.91	2.176	0.114

Data are expressed as the mean ± standard deviation. B = Bone, B.Ar = bone area, Tb.Wi = trabecular width, Tb.Sp = trabecular separation. Different letters in columns indicate statistical difference among groups after a Tukey's Post Hoc Test. Significance level was set at 5% (p<0.05).

**Fig 1:**
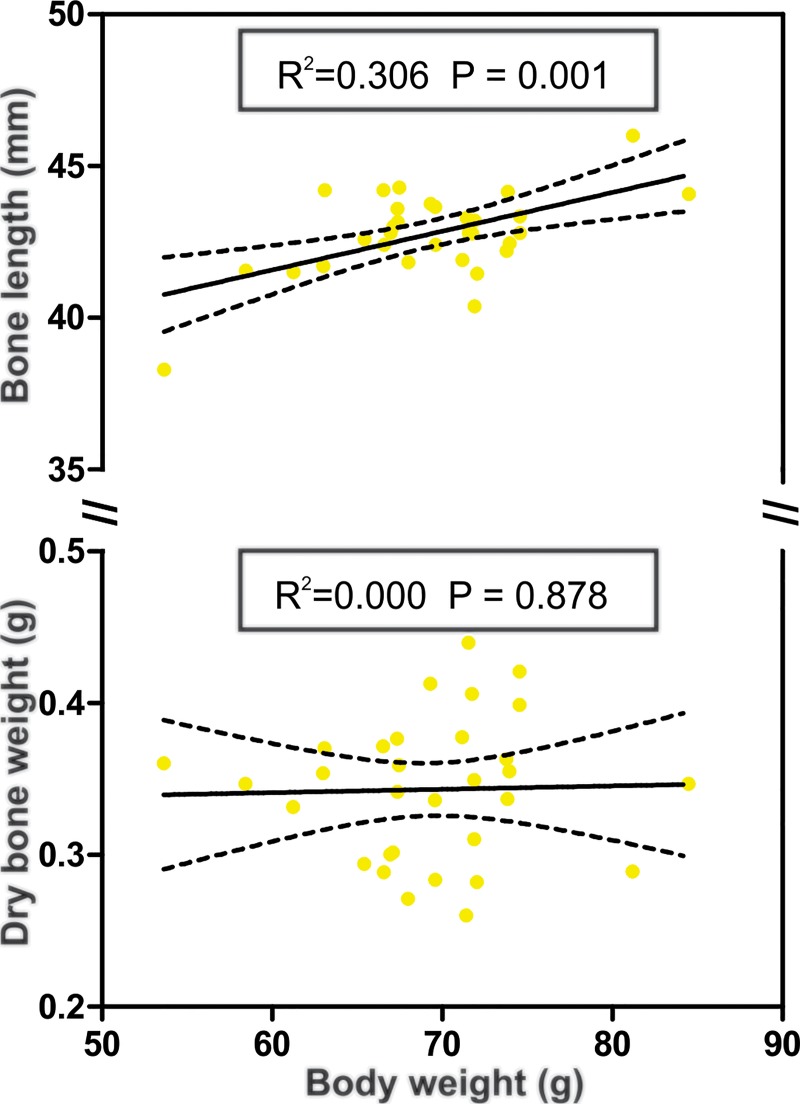
Correlations of body weight with bone characteristics in *Artibeus lituratus* females. Correlation between body weight and bone length (top) and between body weight and dry bone weight (bottom). Black lines represent regression line and dotted lines respresent lower and upper confidence limit (95%).

We found a positive relationship between body weight and bone length following a liner regression (R^2^ = 0.306, p = 0.001), although body weight and bone dry weight showed no significant relationship (Fig 1).

Regarding the biochemical parameters, calcium and phosphorous contents did not present any significat differences among reproductive groups (Table 2). Considering the biomechanical properties, maximum load at fracture and maximum displacement were similar among groups (Table 2).

**Table 2:** Biochemical and bone strength parameters of reproductive female bats (*Artibeus lituratus*).

	Reproductive groups					
	Non-pregnant	Pregnancy	Lactation	Post-lactation	F	*P*
Bone calcium (mg/g)	9.35 ± 1.29	8.64 ± 1.04	8.53 ± 0.95	10.13 ± 0.79	0.109	0.954
Bone phosphorous (mg/g)	0.32 ± 0.03	0.30 ± 0.02	0.31 ± 0.02	0.36 ± 0.03	0.251	0.86
Maximum load fracture (N)	38.80 ± 10.13	44.28 ± 7.34	40.74 ± 8.6	47.32 ± 9.09	1.319	0.289
Maximum displacement (mm)	1.58 ± 0.35	1.63 ± 0.21	1.42 ± 0.16	1.33 ± 0.17	2.317	0.098
Bone stiffness (N/mm)	40.70 ± 13.07	44.22 ± 7.52	44.18 ± 9.12	48.89 ± 11.18	0.732	0.052

Data are expressed as mean ± standard deviation. N = Newton. Significance level was set at 5% (P<0.05).

**Fig 2:**
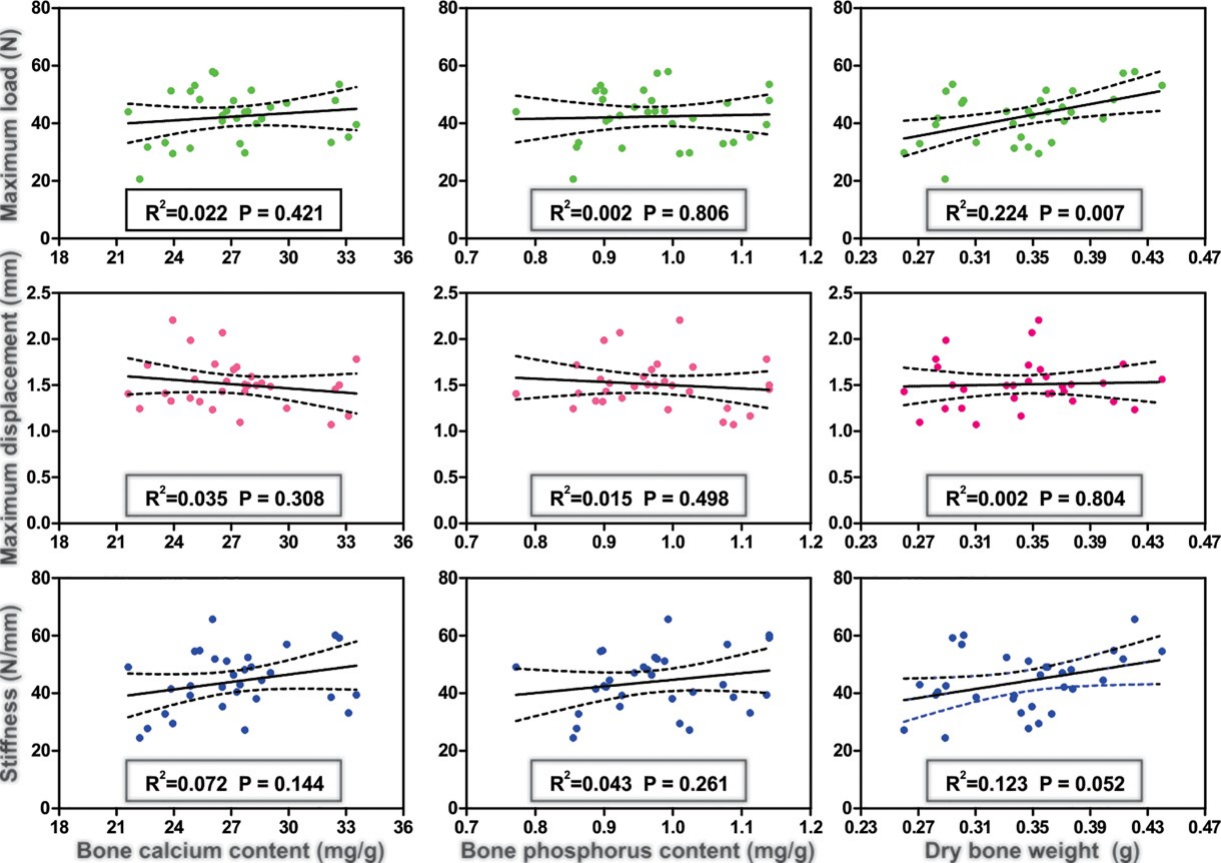
Correlations between bone characteristics in *Artibeus lituratus* females. Correlation between bone calcium, bone phosphorus and dry bone weight with bone strength parameters in *Artibeus lituratus* females. Black lines represent regression line and dotted lines represent 95% confidence limit.

Bone mineral content was not correlated to bone strength parameters, and only bone dry weight was positively correlated to maximum load (R^2^ = 0.224, p = 0.007) (Fig 2).

**Fig 3:**
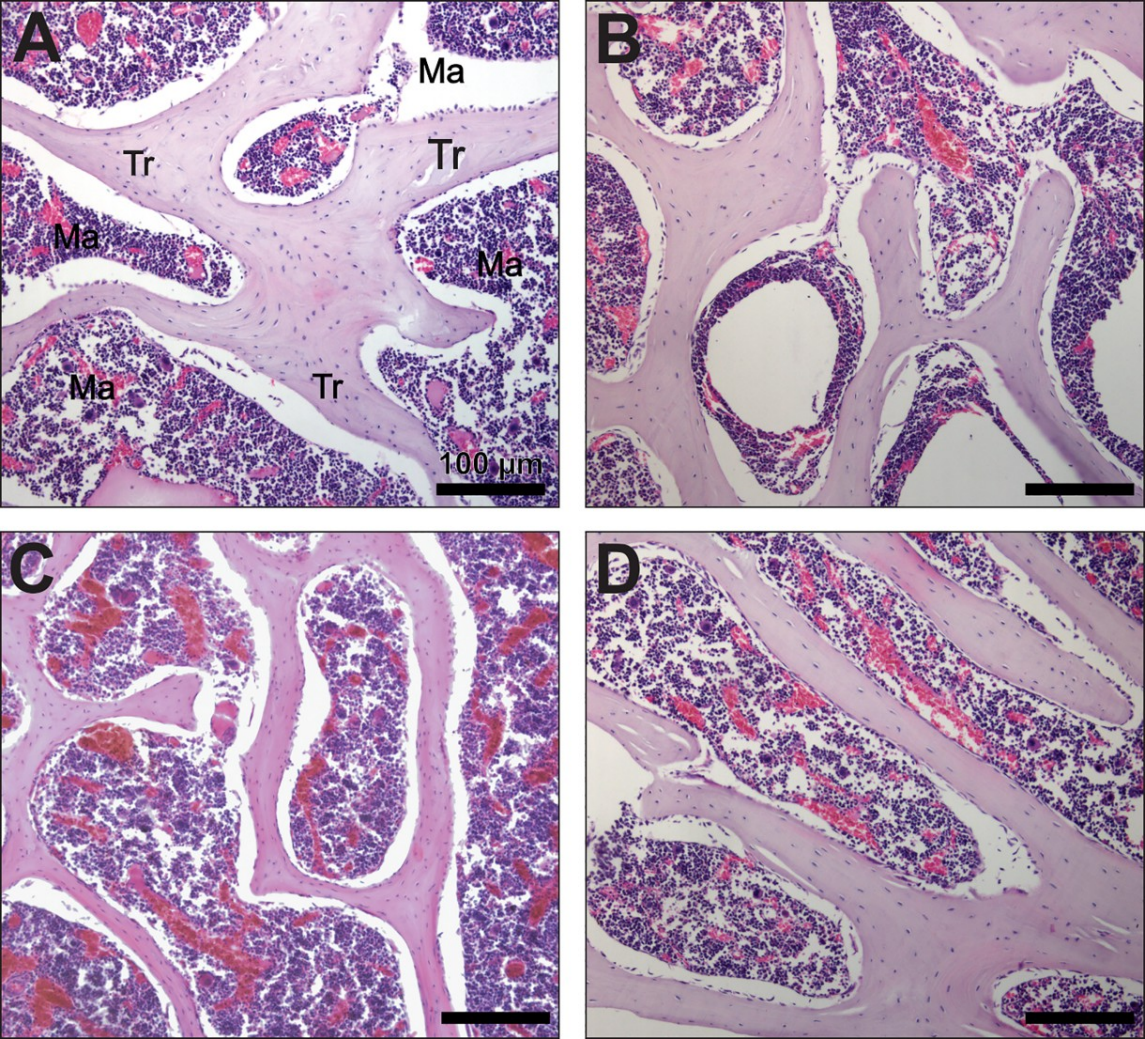
Bone histology of *Artibeus lituratus* females. Representive areas of trabecular bone in Non-pregnant (A), pregnant (B), lactating (C) and post-lactating (D) female *Artibeus lituratus*. Note that trabeculae are thinner in lactating female. Tr = trabecula, Ma = bone marrow. Sections were stained with hematoxylin and eosin.

Results presented here are corroborated by the fact that lactating females showed decreased trabecular bone area when compared to pregnant and post-lactating females (Fig 3).

## Discussion

This study reports, for the first time, bone loss during lactation in a fruit-eating bat. Bone loss was confirmed by decreased trabecular area in the epiphysis of the humerus. Although confirmed, this loss seems to be marginal due to the lack of changes in other parameters, such as trabecular width and trabecular separation. The hypothesis that reproduction is a period of high risk of bone fracture in female bats was not supported for this species, since bone strength parameters were not decreased in pregnant or lactating females. In addition, our findings suggest that the strength of the humerus in *A*. *lituratus* may be more influenced by other bone components rather than calcium or phosphorus. Connective tissue components such as collagen and elastin can influence force production during maximal effort, act on stability, responsiveness and also help maintaining bone strength [29].

Calcium demand is higher during lactation compared to pregnancy, in order to supply the required calcium to the newborn at high rates [30]. The main physiological mechanism maintaining maternal calcium homeostasis during lactation involves hormones such as prolactin and parathyroid hormone-related protein (PTHrP), which induce bone resorption and calcium release by osteoclasts [31,32].

These hormones cause a decrease in bone tissue, mainly at trabecular areas [33]. In our study, the pattern of bone loss found suggests that this same mechanism is active in *A*. *lituratus*, since lactating bats showed reduced trabecular areas compared to pregnant and post-lactating bats, similarly to the pattern observed in insectivorous bats [10,11].

Studies in rats [34], cows [35], bats [9] and humans [36] have shown that dietary calcium supplementation was not enough to sufficiently prevent bone loss during lactation, suggesting that it may be an inherent physiological consequence of this period, probably due to the fact that the physiological environment of lactation is incompatible with bone calcium retention [14]. Although calcium supplementation alone cannot eliminate bone loss, increased dietary calcium intake is proven to reduce the problem [34–37]. The great fruit-eating bat feeds preferentially on figs [38] and occasionally consumes leaves throughout the year, both rich in calcium [39–41], which suggests that reproductive females probably do not have limited or seasonal access to dietary calcium. The easy access to this mineral source can justify our findings, corroborating that bone loss in lactating females was not extensive enough to alter other bone parameters such as trabecular width, trabecular separation or minerals content.

In addition to access to calcium-rich food items, other mechanisms may play a role to reduce the effect of pregnancy and lactation on bone mass and bone strength parameters in locomotion-related bones such as the humerus. Bone resorption, for instance, may take place mostly in other bones like vertebrae, mandible or ribs, thus decreasing the pressure on the wing bones. Despite the fact that resorption occurs simultaneously at different bones in bats [10], the its extent in each bone is still unknown.

Another fact to be considered is that an increase in bone formation and a decrease in bone resorption can rapidly overcome bone loss incurred during lactation in post-lactating female mammals [42,43]. This positive change in bone turnover is supported by our findings, since post-lactating females showed higher values of trabecular bone area compared to lactating bats, suggesting that *A*. *lituratus* shares a similar pattern for bone recovery with other terrestrial mammalian species [44].

Bone tissue is formed by an inorganic portion (i.e. minerals) and an organic portion, constituted mostly by type I collagen. The mineral portion gives most of the strength and stiffness to the bone [12,45], so a decrease in this component is usually associated with a decrease in bone resistance to fractures in terrestrial mammals [46]. We did not observe any significant changes in whole-bone calcium or phosphorous content among *A*. *lituratus* reproductive groups, or any relationship between mineral content and bone strength parameters. However, we found a positive correlation between bone dry weight and maximum load at fracture, suggesting that organic components are highly important to bone strength as compared to mineral content itself. Long bones of bat wings are less mineralized than long bones of non-flying mammals [47], which in addition to a proximodistal gradient of decreasing mineralization in wing bones [15], provides the wing with enough elasticity and low flexural stiffness to hold high bending strains during flight [47].

To be considered a physiological cost of reproduction, bone loss should decrease the probabilities of survival during pregnancy and lactation, rising a conflict between increasing litter size and maintaining skeletal integrity. The evidence gathered from the humerus of *A*. *lituratus* reproductive females did not support this idea, although studies on other bat species that deliver more than one offspring at a time (e.g. *Lasiurus*), or species with low dietary calcium contents (e.g. nectarivorous bats), or studies testing other bones or regional differences within bones, may result in new evidence that may improve our understanding on the role of calcium and bone turnover in bats’ life history. It is important to take into consideration when interpreting the results that this study was performed on wild bats, with unavoidable individual differences in age, nutritional and health histories; in addition to a small sample size associated to the use of wildlife species, which influenced the statistical significance of some parameters tested. However, as some patterns are consistent with other studies, we believe that our results reflect the physiology of this Neotropical frugivorous bat.

## Supporting information

S1 Fig H&E: Histological section of trabecular bone stained with Hematoxylin and eosin. B&W: Black (trabeculae) and white (background) images of trabecular bone. Tb.Wi: Quantification of trabecular width by the sphere fitting method in the white and black images. Tb.Sp: Quantification of trabecular separation by the sphere fitting method in the white and black images. NR = non-reproduction, PR = pregnancy, LA = lactation, PL = post-lactation.
(TIF)

S1 File Results of Shapiro-Wilk and Levene’s tests. (DOCX)

S2 File Full dataset. (XLSX)
