# Supplementary figures and images for: Is bone loss a physiological cost of reproduction in the Great fruit-eating bat Artibeus lituratus?
Source: PLoS One. 2019 Mar 28;14(3):e0213781. doi: 10.1371/journal.pone.0213781 (PMC6438481; doi:10.1371/journal.pone.0213781)

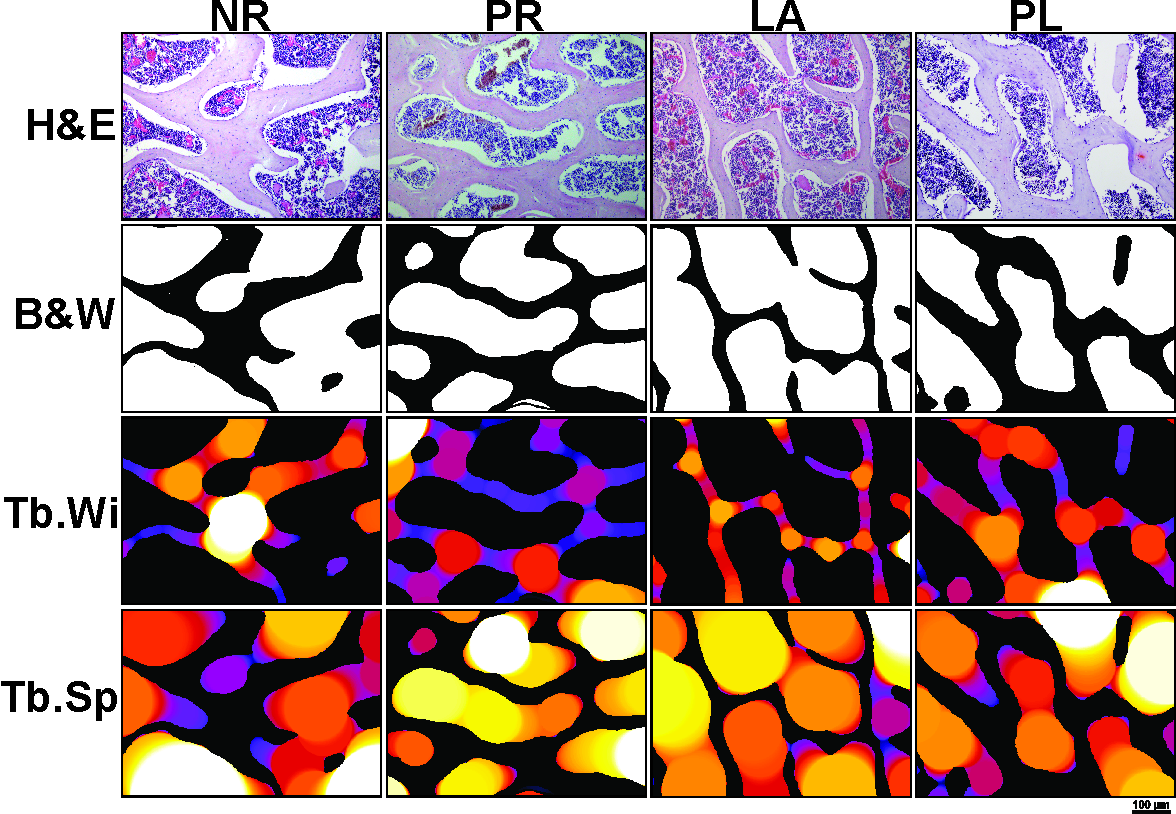

Supplement: S1 Fig — B&W: Black (trabeculae) and white (background) images of trabecular bone. Tb.Wi: Quantification of trabecular width by the sphere fitting method in the white and black images. Tb.Sp: Quantification of trabecular separation by the sphere fitting method in the white and black images. NR = non-reproduction, PR = pregnancy, LA = lactation, PL = post-lactation. (TIF) [file pone.0213781.s001.tif]
