# Supplementary material for: Is bone loss a physiological cost of reproduction in the Great fruit-eating bat Artibeus lituratus?
Source: PLoS One. 2019 Mar 28;14(3):e0213781. doi: 10.1371/journal.pone.0213781 (PMC6438481; doi:10.1371/journal.pone.0213781)
Supplement: S1 File — (DOCX) [file pone.0213781.s002.docx]

| **Variable** | **N** | **Shapiro-Wilk** | | **Levene** | |
| --- | --- | --- | --- | --- | --- |
|  |  | **W** | **p** | **F** | **p** |
| Body_weight_g | 31 | 0.97 | 0.8143 | 1.8134 | 0.1685 |
| Bone_length_mm | 31 | 0.96 | 0.6853 | 1.0975 | 0.3672 |
| Dry_bone_g | 31 | 0.95 | 0.4012 | 0.9688 | 0.4218 |
| Wet_bone_g | 31 | 0.97 | 0.8103 | 0.0657 | 0.9776 |
| Bone_water_content_% | 31 | 0.98 | 0.9178 | 0.9967 | 0.4094 |
| Trabecular_bone _area_μm2 | 31 | 0.92 | 0.1112 | 0.8141 | 0.4973 |
| Trabecular_width_µm | 31 | 0.97 | 0.8054 | 0.9082 | 0.4501 |
| Trabecular_separation_µm | 31 | 0.97 | 0.8518 | 0.4823 | 0.6974 |
| Bone_calcium_content_mg*g-1 | 31 | 0.93 | 0.1556 | 0.8343 | 0.4868 |
| Phosphorous_bone_content_mg*g-1 | 31 | 0.95 | 0.3484 | 0.6727 | 0.5762 |
| Maximum_load_at_fracture _N | 31 | 0.96 | 0.5707 | 0.7532 | 0.5301 |
| Maximum_displacement_at_fracture_mm | 31 | 0.94 | 0.2639 | 2.6542 | 0.0687 |
| Bone_stiffness_N*mm-1 | 31 | 0.97 | 0.7625 | 1.4467 | 0.2512 |

**S1 File.** Results of Shapiro-Wilk and Levene’s tests.
